# Adult-onset Bartter syndrome type IV B with ACTH secreting pituitary microadenoma

**DOI:** 10.1590/2175-8239-JBN-2024-0204en

**Published:** 2025-02-24

**Authors:** Gerry George Mathew

**Affiliations:** 1 SRM Medical College Hospital and Research Centre, Department of Nephrology, Chengalpattu, India.

Dear Editor,

We present the case of a 38-year-old female patient who presented to our hospital with a history of vertigo, abdominal distension, weakness in all limbs, and facial edema for several days. She had a history of recurrent quadriparesis and polyuria over the previous 3 years. Additionally, she had new-onset type 2 diabetes mellitus (T2DM) over the past 2 years, was normotensive with a blood pressure of 100/64 mmHg, and euvolemic with cushingoid facies. Initial evaluation revealed a mild emotional lability and proximal muscle weakness in all limbs. Labs revealed serum potassium levels of 1.9 meq/litre, serum magnesium of 2.4 meq/litre, serum sodium of 128 meq/litre, serum chloride of 90 meq/litre, serum bicarbonate of 34 with normal renal function. ABG showed pH 7.51, pC0_2_ 44.6, and bicarbonate level of 35.8, which was indicative of metabolic alkalosis. With regard to metabolic alkalosis, normotension, hypokalaemia, a urine spot potassium creatinine ratio of 2.6 and a urine spot calcium creatinine ratio of 0.95 were found, which was indicative of kaliuresis and hypercalciuria, thereby confirming adult-onset Bartter syndrome. Abdomen and chest computed tomography findings were normal and non-contributory. In view of the emotional lability, elevated glucose levels, and cushingoid facies, 8 am serum cortisol was measured and showed a value of 56.5 mcg/dL (range 5–25 mcg/dL) and serum adrenocorticotropic hormone (ACTH) level was 138 pg/mL (range 10–60 pg/ml). Brain MRI revealed pituitary microadenoma in the posterior pituitary gland (Figure 1). The patient was treated with oral and intravenous potassium chloride, spironolactone, and indomethacin. Subsequently, the patient underwent transsphenoidal surgery for pituitary microadenoma, which was found to be an ACTH-secreting pituitary microadenoma. Genetic testing results confirmed the diagnosis of autosomal recessive Bartter syndrome type 4b, attributed to recessive digenic mutations in both CLCNKA and CLCNKB genes (1p36.13). On audiological examination, the patient had mild sensorineural hearing loss. On follow-up after 3 months, the patient was stable with normal potassium levels, and was free of emotional lability and confusion.

**Figure 1:**
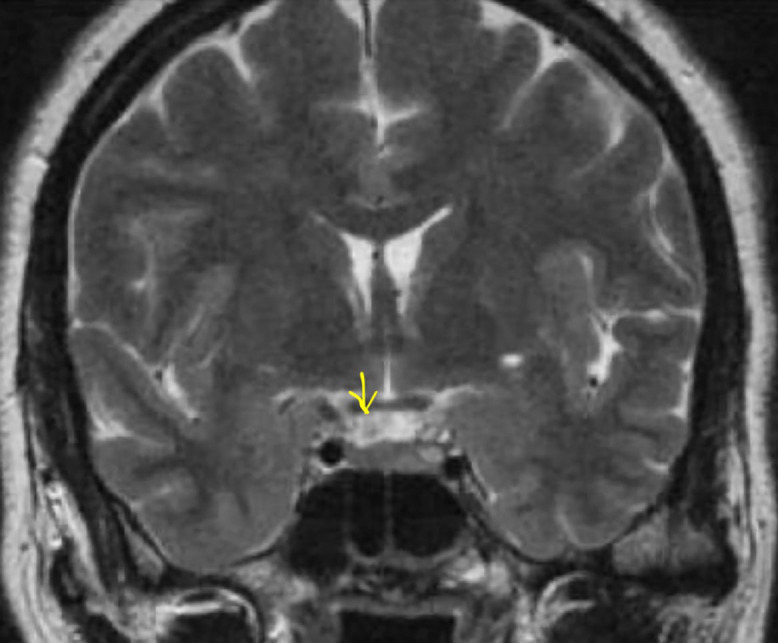
T2 weighted magnetic resonance imaging of brain with pituitary cuts showing isointense lesion (yellow arrow) in the pituitary gland suggestive of pituitary microadenoma (Magnification:300 DPI).

Bartter syndrome (BS) is a genetic disorder involving renal salt wasting, premature birth, polyhydramnios, hypokalemia, normotension, and hypochloremic metabolic alkalosis^1^. It includes five subtypes, most with autosomal recessive inheritance^1^. These mutations cause renal salt wasting, renin-angiotensin system activation, altered tubuloglomerular feedback, and increased prostaglandin synthesis due to hyperactive cyclooxygenases (COX)^2^. Type 4B features recessive a digenic mutation in CLCNKA and CLCNKB genes, manifesting as severe, prenatal disease^1,2^. This mutation also causes sensorineural deafness due to the critical role of the CLCNKA gene in endolymph potassium regulation^1,2^. This case is unique due to the fact that is is an adult-onset Bartter’s syndrome with CLCNKA and CLCNKB mutations alongside an ACTH-secreting pituitary microadenoma, which has not been previously documented. All clinical and laboratory features were consistent with Bartter’s syndrome, but the late onset posed a diagnostic challenge. Treatment includes potassium supplementation, potassium-sparing diuretics, COX inhibitors like indomethacin, and angiotensin receptor blockers, as appropriate^2^.

ACTH-secreting pituitary tumors are the leading cause of endogenous hypercortisolism^3^. It has symptoms such as muscle weakness, emotional instability, facial plethora, psychiatric and neurocognitive changes, thin skin, high blood glucose levels, and pronounced cushingoid facies^4^. In our patient, hypokalemia with hypercortisolism worsened muscle weakness, and recurrent quadriparesis may have resulted from uncorrected hypokalemia. The size of the tumor does not correlate with hypercortisolism severity, and ACTH-secreting pituitary microadenomas are more common in Cushing’s disease than macroadenomas^4^. Management includes initial surgical resection via the transsphenoidal approach, yielding an 80% cure rate^4^. If surgery fails, radiation and chemotherapy are options^4^. The patient’s normotension, despite metabolic and electrolyte disturbances due to ACTH excess and hypercortisolism, prompted genetic evaluation, confirming underlying type 4B Bartter’s syndrome.

The patient also developed diabetes mellitus alongside Bartter’s syndrome. Persistent hypokalemia is known to cause glucose intolerance^3^, and studies indicate that mildly to moderately low serum potassium levels contribute to diabetes mellitus^3^. This case posed a unique management challenge and is the first reported instance of Bartter’s syndrome type 4B with an associated ACTH-secreting pituitary microadenoma.

## Data Availability

The data for substantiating the findings of this manuscript are available with the corresponding author and can be made available on request.
