# Supplementary material for: Adult-onset Bartter syndrome type IV B with ACTH secreting pituitary microadenoma
Source: J Bras Nefrol. 2025 Feb 24;47(3):e20240204. doi: 10.1590/2175-8239-JBN-2024-0204en (PMC11849558; doi:10.1590/2175-8239-JBN-2024-0204en)
Supplement: Translation

# Translation: Síndrome de Bartter tipo IV B de início na idade adulta com microadenoma hipofisário secretor de ACTH

**DOI:** 10.1590/2175-8239-JBN-2024-0204pt

**Authors:** Gerry George Mathew

**Affiliations:** 1 SRM Medical College Hospital and Research Centre, Department of Nephrology, Chengalpattu, India.

Prezado Editor,

Apresentamos o caso de um paciente do sexo feminino, 38 anos, que deu entrada em nosso hospital com histórico de vertigem, distensão abdominal, fraqueza em todos os membros e edema facial por vários dias. A paciente apresentava histórico de quadriparesia e poliúria recorrentes nos últimos 3 anos. Além disso, apresentava diabetes mellitus tipo 2 (DM2) de início recente nos últimos 2 anos, era normotensa com pressão arterial de 100/64 mmHg e euvolêmica com fácies cushingoide. A avaliação inicial revelou leve labilidade emocional e fraqueza muscular proximal em todos os membros. Os exames laboratoriais revelaram níveis séricos de potássio de 1,9 meq/litro, magnésio sérico de 2,4 meq/litro, sódio sérico de 128 meq/litro, cloreto sérico de 90 meq/litro, bicarbonato sérico de 34 com função renal normal. A gasometria arterial (GA) revelou pH de 7,51, pC02 de 44,6 e nível de bicarbonato de 35,8, indicativos de alcalose metabólica. Em relação à alcalose metabólica, verificou-se normotensão, hipocalemia, relação potássio/creatinina de 2,6 na urina isolada e relação cálcio/creatinina de 0,95 na urina isolada, indicativos de caliurese e hipercalciúria, confirmando assim a síndrome de Bartter de início na idade adulta. Os achados da tomografia computadorizada de abdômen e tórax foram normais e não contributivos. Em virtude da labilidade emocional, níveis elevados de glicose e fácies cushingoide, foi realizada a medição do cortisol sérico às 8 horas da manhã, que apresentou um valor de 56,5 mcg/dL (intervalo de 5–25 mcg/dL) e o nível sérico do hormônio adrenocorticotrópico (ACTH) foi de 138 pg/mL (intervalo de 10–60 pg/ml). A ressonância magnética do crânio revelou microadenoma hipofisário na glândula pituitária posterior (Figura 1). A paciente foi tratada com cloreto de potássio oral e intravenoso, espironolactona e indometacina. Posteriormente, a mesma foi submetida a uma cirurgia transesfenoidal para tratamento de um microadenoma hipofisário, que se revelou um microadenoma hipofisário secretor de ACTH. Os resultados dos testes genéticos confirmaram o diagnóstico de síndrome de Bartter tipo 4b autossômica recessiva, atribuída a mutações digênicas recessivas nos genes CLCNKA e CLCNKB (1p36.13). No exame audiológico, a paciente apresentou perda auditiva neurossensorial leve. No acompanhamento após três meses, ela se manteve estável, com níveis normais de potássio, e não apresentou labilidade emocional e confusão.

**Figura 1:** Imagem de ressonância magnética ponderada em T2 do cérebro com cortes pituitários mostrando lesão isointensa (seta amarela) na glândula pituitária, sugestiva de microadenoma hipofisário (Ampliação: 300 DPI).

A síndrome de Bartter (SB) é uma condição genética que envolve perda renal de sal, parto prematuro, polidrâmnio, hipocalemia, normotensão e alcalose metabólica hipoclorêmica^1^. A síndrome inclui cinco subtipos, a maioria com herança autossômica recessiva^1^. Essas mutações causam perda renal de sal, ativação do sistema renina-angiotensina, alteração no feedback tubuloglomerular e aumento da síntese de prostaglandinas devido à hiperatividade das ciclooxigenases (COX)^2^. O tipo 4B caracteriza-se por uma mutação digênica recessiva nos genes CLCNKA e CLCNKB, manifestando-se como doença pré-natal grave^1,2^. Essa mutação também causa perda auditiva neurossensorial devido ao papel fundamental do gene CLCNKA na regulação do potássio na endolinfa^1,2^. Este caso é único por se tratar de uma síndrome de Bartter de início na idade adulta com mutações CLCNKA e CLCNKB juntamente com um microadenoma hipofisário secretor de ACTH, algo não documentado anteriormente. Todas as características clínicas e laboratoriais foram consistentes com a síndrome de Bartter, mas o início tardio representou um desafio diagnóstico. O tratamento inclui suplementação de potássio, diuréticos poupadores de potássio, inibidores de COX - como a indometacina – e bloqueadores dos receptores de angiotensina, conforme apropriado^2^.

Tumores hipofisários secretores de ACTH são a principal causa de hipercortisolismo endógeno^3^. Apresenta sintomas como fraqueza muscular, instabilidade emocional, pletora facial, alterações psiquiátricas e neurocognitivas, pele fina, níveis elevados de glicose no sangue e fácies cushingoide acentuada^4^. Em nossa paciente, a hipocalemia com hipercortisolismo agravou a fraqueza muscular, e a quadriparesia recorrente pode ter resultado da hipocalemia não corrigida. O tamanho do tumor não se correlaciona com a gravidade do hipercortisolismo, e os microadenomas hipofisários secretores de ACTH são mais comuns na doença de Cushing do que os macroadenomas^4^. O manejo inclui a ressecção cirúrgica inicial por meio da abordagem transesfenoidal, resultando em uma taxa de cura de 80%^4^. Se a cirurgia falhar, opções como radioterapia e quimioterapia são possíveis^4^. A normotensão da paciente, apesar dos distúrbios metabólicos e eletrolíticos devido ao excesso de ACTH e ao hipercortisolismo, motivou a avaliação genética, confirmando a síndrome de Bartter tipo 4B subjacente.

A paciente também desenvolveu diabetes mellitus juntamente com a síndrome de Bartter. Sabe-se que a hipocalemia persistente pode causar intolerância à glicose^3^ e estudos indicam que níveis séricos de potássio leves a moderadamente baixos contribuem para o diabetes mellitus^3^. Esse caso representou um desafio único de manejo e é o primeiro caso relatado de síndrome de Bartter tipo 4B com um microadenoma hipofisário secretor de ACTH associado.

## Data Availability

Os dados que fundamentam os achados deste manuscrito estão disponíveis com o autor correspondente e podem ser disponibilizados mediante solicitação.
